# Mapping evidence on knowledge of breast cancer screening and its uptake among women in Ghana: a scoping review

**DOI:** 10.1186/s12913-022-07775-z

**Published:** 2022-04-20

**Authors:** Agani Afaya, Abdul-Aziz Seidu, Somin Sang, Vida Nyagre Yakong, Richard Adongo Afaya, Jinhee Shin, Bright Opoku Ahinkorah

**Affiliations:** 1 College of Nursing, Yonsei University, 50-1, Yonsei-ro, Seodaemun-gu, Seoul, 03722 South Korea; 2 Department of Nursing, School of Nursing and Midwifery, University of Health and Allied Sciences, Ho, Ghana; 3 College of Public Health, Medical and Veterinary Sciences, James Cook University, Douglas, Australia; 4 Faculty of Built and Natural Environment, Department of Estate Management, Takoradi Technical University, Takoradi, Ghana; 5 Centre for Gender and Advocacy, Takoradi Technical University, P.O.Box 256, Takoradi, Ghana; 6 Department of Preventive Health Nursing, School of Nursing and Midwifery, University for Development Studies, Tamale, Ghana; 7 Department of Midwifery and Women’s Health, School of Nursing and Midwifery, University for Development Studies, Tamale, Ghana; 8 Mo-Im Kim Nursing Research Institute, College of Nursing, Yonsei University, Seoul, South Korea; 9 School of Public Health, Faculty of Health, University of Technology Sydney, Sydney, Australia

**Keywords:** Breast cancer, screening, Ghana, Scoping review

## Abstract

**Introduction:**

Female breast cancer is currently the most commonly diagnosed cancer globally with an estimated 2.3 million new cases in 2020. Due to its rising frequency and high mortality rate in both high- and low-income countries, breast cancer has become a global public health issue. This review sought to map literature to present evidence on knowledge of breast cancer screening and its uptake among women in Ghana.

**Methods:**

Five databases (PubMed, CINAHL*,* PsycINFO, Web of Science, and EMBASE) were searched to identify relevant published studies between January 2012 and August 2021 on knowledge of breast cancer screening and its uptake among women. The Preferred Reporting Items for Systematic Reviews and Meta-Analyses (PRISMA) extension for scoping reviews and the six-stage model by Arksey and O’Malley were used to select and report findings.

**Results:**

Of the 65 articles retrieved, 14 records were included for synthesis. The review revealed varied knowledge levels and practices of breast cancer screening among women across a few regions in Ghana. The knowledge level of women on breast cancer screening was high, especially in breast cancer screening practice. Breast cancer screening practice among women was observed to be low and the most identified barriers were lack of technique to practice breast self-examination, having no breast problem, lack of awareness of breast cancer screening, and not having breast cancer risk. The results further showed that good knowledge of breast cancer screening, higher educational level, increasing age, physician recommendation, and household monthly income were enabling factors for breast cancer screening uptake.

**Conclusion:**

This review showed varied discrepancies in breast cancer screening uptake across the regions in Ghana. Despite the benefits of breast cancer screening, the utilization of the screening methods across the regions is very low due to some varied barriers from the different regions. To increase the uptake of breast cancer screening, health workers could employ various strategies such as community education and sensitization on the importance of breast cancer screening.

**Supplementary Information:**

The online version contains supplementary material available at 10.1186/s12913-022-07775-z.

## Introduction

Breast cancer is currently a global public health problem due to its increasing prevalence coupled with the high mortality rate among women in both high-income and low-income countries [1]. Female breast cancer is currently the most commonly diagnosed cancer in the world, with an estimated 2.3 million new cases in 2020 and the fifth leading cause of cancer-related mortality worldwide, with 685,000 deaths [1]. Between 1990 and 2017, it was estimated that the global breast cancer cases increased by about 123.14% [2]. The GLOBOCAN cancer prediction tool estimates that by 2040 the global incidence of breast cancer cases is expected to increase more than 46% [3]. In 2012, sub-Saharan Africa (SSA) recorded about 94,378 breast cancer cases for the first time [4]. It is estimated that by 2050 the prevalence of breast cancer cases in SSA will double [5].

Globally, SSA has the highest mortality of breast cancer [1] and with less than 40% five-year survival rate, compared to high-income countries such as the United States with an 86% survival rate [4]. Whereas breast cancer mortality in many high-income countries has seen a significant decrease over the past 25 years due to increases in awareness, early detection, and treatments, it is now the leading cause of death from cancer in low-middle-income countries (LMICs) [6]. Also, mortality rates in SSA have seen an exponential increase and ranked among the world’s highest exposing the weaker health infrastructure and poor survival outcomes [1]. The low survival rates are relatively attributed to the late-stage presentation of breast cancer. Evidence from 83 studies across 17 countries in SSA revealed that 77% of all staged cases were diagnosed at stage III/IV [7] contributing to the burden in the sub-region. Because there are lack/inadequate population-based mammography screening programs in low-resource settings, efforts should be targeted at promoting early detection through breast cancer awareness, breast self-examination (BSE), and clinical breast examination (CBE) by skilled health providers, [8, 9] followed by timely and appropriate interventions to improve the survival rate in LMICs [1]. A recent study among five countries in SSA estimated that 28 to 37% of breast cancer mortalities could be prevented through earlier diagnosis and adequate treatment [10].

According to the WHO - Cancer Country Profile of Ghana 2020, breast cancer is the number one cancer among women in Ghana with an incidence of 20.4% with a relatively high mortality rate [11]. The effective way of detecting breast cancer is through regular screening [12]. Despite breast cancer screening (BCS) benefits, the utilization of BCS services is relatively low in Ghana as compared to some high-income countries [13]. A recent study conducted in Ghana revealed a relatively low BCS prevalence of 4.5% among older women [13].

Factors influencing participation in BCS services vary differently across the regions in Ghana. A recent study conducted in Ghana revealed that age, education, ethnicity, income quantile, father’s education, mother’s employment, and chronic disease status were associated with the uptake of BCS practices [14]. Another study within the country identified that accessibility and affordability of BCS services were associated with BCS uptake [15].

Hence, Ghana needs immediate action to promote early detection of breast cancer through the various screening methods as these efforts could help achieve the Sustainable Development Goal (SDG) agenda 3.4 by 2030 [16]. To promote early detection of breast cancer, women’s knowledge, attitude, and practice of BCS services are essential [17]. Evidence indicates that having knowledge of BCS practices has a positive impact on the prevention and early detection of breast cancer [17, 18]. Therefore, women’s knowledge of BCS may also have a positive influence on the attitude and practice of BCS services.

At the time of conducting this study and to the best of our knowledge, no review has comprehensively explored women’s knowledge and BCS practices in Ghana. Although a recent similar review has been conducted in SSA [17], it is important to consider and focus on the Ghanaian context. Hence, a broad perspective of understanding women’s knowledge and practice of BCS is critical to help design effective public health strategies and interventions to improve the uptake of BCS in Ghana. Thus, this review aimed to comprehensively and systematically map literature and describe the evidence on women’s knowledge and practice of BCS in Ghana.

### Definitions

The Authors defined knowledge, attitude, and practice based on the framework of the World Health Organization *Guide to Developing Knowledge, Attitude, and Practice Surveys* [19].

*Knowledge:* refers to the general knowledge of the various methods of breast cancer screening being utilized by Ghanaian women to enhance early detection, diagnosis, and treatment.

*Attitude:* refers to women’s perception or feeling or opinion about the various breast cancer screening methods to ensure early detection, diagnosis, and treatment of breast cancer.

*Practice:* referred to the actions undertaken by Ghanaian women to utilize the various breast cancer screening methods (BSE, CBE, Mammography) to ensure early detection diagnosis and treatment of breast cancer.

## Methods

This scoping review used the six stages of Arksey and O’Malley’s framework outlined in the Joanna Briggs Institute manual [20]. The Arksey and O’Malley framework [21] includes these six stages (a) identifying the research question, (b) identification of relevant studies, (c) selection of studies, (d) charting data, (e) collating, summarizing, and reporting evidence. The authors did not conduct the optional stage of consultation with stakeholders [21, 22] because of our background in the subject area. Also, this study was reported following the Preferred Reporting Items for Systematic Reviews and Meta-Analyses extension for scoping reviews (PRISMA-ScR) checklist [23] (Supplementary file 1).

### Phase 1: Identifying research questions

The identification of research questions ensures the linkage and clarification of the study purpose and research questions [20]. The primary review question is: what evidence exists on women’s knowledge and practice of BCS in Ghana?

The following are the sub-review questions: (1) what is the evidence on the knowledge of BCS among women in Ghana? (2) what is the evidence on the practice of BCS among women in Ghana? (3) what are the barriers and predictors of BCS practices among women in Ghana?

### Phase 2: Identification of relevant studies

We employed the three-step search strategy proposed by the JBI team of researchers for all types of reviews [20]. Step one ensured an initial limited search for already existing published research articles on knowledge and practice of BCS among women in Ghana. The initial limited search for potentially eligible articles was conducted in the following databases PubMed and CINAHL via EBSCO*host.* The initial limited search ensured the identification of relevant keywords to be used. Step two involved a formal search after finalizing and combining the following keywords (‘breast cancer screening’, mammography OR mammogram, ‘breast self-examination’, ‘clinical breast examination’ Knowledge and practice) using the Boolean operators. The formal comprehensive and exhaustive search was conducted in the following databases: PubMed, CINAHL via EBSCO*host,* PsycINFO, Web of Science, and Embase. Step three adopted the snowballing technique which involved manually tracing the reference list of identified relevant studies for additional studies. This was done up to the point of saturation where no new information emanates from the subsequent manual search of articles. The detailed search strategy is shown in Table S1 in the Appendix.

### Phase 3: Selection of studies

Studies included in this review were selected based on the following criteria: (a) primary studies conducted in Ghana (b) published between January 2012 to August 2021 (c) studies reporting evidence on women 18 years and above, (d) reporting evidence on knowledge and practice of BCS among women (e) articles published in the English language. The exclusion criteria were based on: (a) studies conducted in other countries but not in Ghana, (b) Studies reporting evidence in men (c) studies published before 2012 (d) studies published in the form of reports, editorials, conference paper, book chapters, and reviews (e) studies involving other cancers.

Two authors (AA and RAA) independently screened the initial titles and abstracts of the retrieved articles for relevance and inclusion. The articles that did not meet the inclusion criteria were excluded. The full-text evaluation was conducted among the remaining articles for inclusion. Where there were disagreements and ambiguity, VNY, AAS, and BOA assessed the article to determine the final set of studies to be included.

### Phase 4: Data charting

We adapted the data extraction form for scoping reviews developed by the JBI researchers to extract data from included studies based on the purpose of this review. Using the data extraction template, two authors (AA and RAA) independently reviewed each article and discussed charted data, and update the data extraction sheet accordingly. Discrepancies and disagreements were resolved by the last author through further adjudication. The data extraction categories included first authors, publication year, study location/context, study type, aim, study population, key findings (knowledge and practice of BCS uptake).

### Phase 5: Collating, summarizing, and reporting the results

The fifth stage of the Arksey and O’Malley methodological framework entails collating, summarizing, and reporting evidence [21]. The Braun and Clarke framework [24] was used to guide the thematic data analysis. The extracted data were read several times to make meaning after which free line by line independent coding was conducted by two authors (AA and RAA). The codes were constantly compared and discussed to resolve differences. The codes were then reviewed, and similar codes were grouped to form themes. A summary report on each theme was presented narratively with the focus on this study’s outcome of interest (knowledge, and practice of BCS).

## Results

### Selection of sources of evidence

**Fig. 1:**
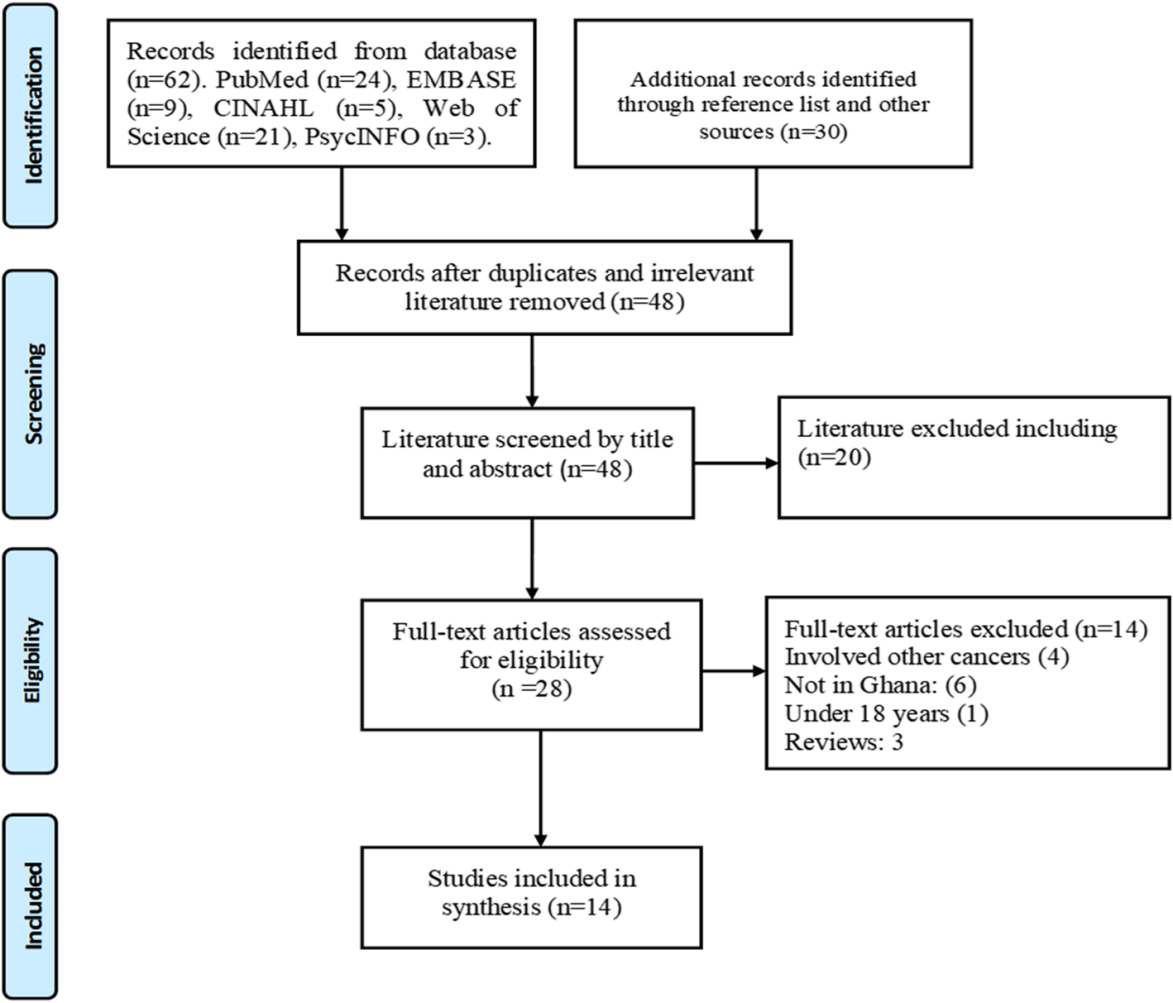
PRISMA flow diagram

A total of 65 articles were retrieved and imported into the endnote reference manager for deduplication. After deduplication, a total of 48 articles were screened for title and abstract. A total of 14 articles [13, 25–37] met the inclusion criteria after a full-text review of 28 articles. See a detailed PRISMA flow diagram (Fig. 1).

### Study characteristics

The cumulative sample size of the 14 studies reviewed comprised 6779 females. Of the 14 studies, 12 studies [13, 25–33, 36, 37] employed descriptive cross-sectional study design while two studies [34, 35] used a mixed-method design. Out of the previous ten regions in Ghana, five studies were conducted in the Greater Accra region followed by three studies in the Ashanti region, two studies in the Volta region, and a study in the Northern region. One study was conducted across two regions (Greater Accra and Brong Ahafo region). Two of the studies were nationwide. Three studies [32, 35, 36] assessed women’s knowledge and practices on BSE, CBE, and mammography while six studies focused on only BSE [26, 28, 30, 31, 34, 37]. Two studies focused on BSE and CBE [27, 33] while one study focused on only mammography [29] (Table 1).

**Table 1:** Characteristics of studies included

Characteristics	Frequency	Reference number
**Region of study**		
National	2	[13, 29]
Greater Accra	5	[25, 26, 31, 32, 34]
Ashanti region	3	[27, 33, 37]
Volta region	2	[30, 36]
Northern region	1	[28]
Greater Accra & Brong Ahafo	1	[35]
**Type of studies**		
Quantitative (cross-sectional)	12	[13, 25–33, 36, 37]
Mixed method (qualitative &quantitative)	2	[34, 35]
**Study setting/location**		
Community setting	8	[13, 25, 29, 30, 33–35, 37]
Hospital setting	2	[27, 32]
University setting	3	[26, 31, 36]
Hospital and community setting	1	[28]
**Screening methods**		
BSE only	6	[26, 28, 30, 31, 34, 37]
MM only	1	[29]
BSE, CBE & MM	3	[32, 35, 36]
BSE & CBE	2	[27, 33]
BSE & MM	1	[25]
BCS (not specific)	1	[13]

*Legend*: *BCS* breast cancer screening, *BSE* breast self-examination, MM: mammography, *CBE* clinical breast examination

### Knowledge of breast cancer screening

Among the 14 studies included, eight studies reported evidence on Ghanaian women’s knowledge of BCS practices [25, 27, 28, 30–32, 34, 37]. Among the various types of BCS, women reported high knowledge of BSE. A study conducted in the Ashanti region reported 95% of female nursing students’ knowledge of BSE, of which 19% of them knew mammography, 15% mentioned CBE, and 60% stated BSE as a screening method for early detection of breast cancer [37]. In the Greater Accra region, Kudzawu et al. [34] reported that 93% of women were aware of BSE. Also, in the Northern region of Ghana, it was reported that 87% of females had knowledge of BSE [28]. Another study in Greater Accra reported that more than three-quarters (76.3%) of the participants knew how to perform BSE [25]. The study by Ghansah [32] reported that 95.4% of women were aware of BSE, 69% were aware of CBE and 54% knew about screening mammography. The study conducted by Dadzi et al. [30] in the Volta region revealed a low level of women’s knowledge on BSE (43%). Participants’ main source of knowledge of BCS was from the media [28, 34, 37], healthcare providers [9, 25, 30, 34], and friends or relatives [30] (see Table 2).

**Table 2:** Summary of study characteristics and findings

First Author (Year)	Location/setting	Study design	Study aim	Sample	Type of BCS	Practices	Knowledge
Agyemang (2020) [13]	National: Community setting.	Cross-sectional study	Estimated the prevalence of and identifying the factors that predict BCS among older adult women in Ghana.	Women (*n* = 2032). Age: > 50 years	No specific type indicated	An estimated 4.5% of women practice BCS. Cervical cancer screening, primary level education were predictors of BCS.	
Amenuke-Edusei (2020) [25]	Greater Accra region: Community setting.	Cross-sectional study	To explore the influence of sociodemographic characteristics, access to healthcare providers, and physicians’ recommendations on Ghanaian women’s BCS practices.	Women (*n* = 194) Age: ≥ 18 years	BSE, and MM	136 (70%) practiced BSE. 82 (42%) practiced BSE once a month. Sixteen (13.7%) had undergone mammography. Routine medical check-up. Barriers to mammography screening included did not have signs of BC, not being aware of breast screening, and not receiving physician recommendations. Physicians’ recommendations, increasing age, and household monthly income were significant predictors of BCS.	More than three-quarters (*n* = 148) knew how to perform BSE. Half (*n* = 99) attributed their knowledge to health care providers.
Boafo (2019) [26]	Greater Accra region: University community setting.	Cross-sectional study	Examined the factors which influence the performance of BSE among female undergraduate students at the University of Ghana.	Female University students (*n* = 308). Age: ≥20 years	BSE	Performance of BSE was associated with good knowledge of BC, BSE perceived barriers, higher self-efficacy.	
Bonsu (2019) [27]	Ashanti region: Hospital setting.	Cross-sectional study	To describe BC knowledge, beliefs, attitudes, and screening efforts by micro-community of advanced BC patients in Ghana.	Women (*n* = 67) Age: ≥18 years	BSE and CBE	Contraceptive use, age at menarche, history of BC, and positive beliefs were associated with BCS.	Half of the participants had positive knowledge of BCS. High knowledge about BC enhanced BSE practices.
Buunaaim (2020) [28]	Northern region: Hospital and community setting.	Cross-sectional study	To assess the knowledge of BC and the practice of BSE among females in the Tamale Metropolis of Northern Ghana.	Women (*n* = 1122). Age: ≥18 years	BSE	Most of the participants, 714 (63.3%), had ever performed BSE. BSE practice increased with increasing reproductive age.	The majority (87%) of the participants had prior knowledge about BSE. The Source of knowledge was from the mass media, health care providers, and friends.
Calys-Tagoe (2020) [29]	National: Community setting.	Cross-sectional study	To determine the uptake of mammography among Ghanaian women aged 40 years or older and to examine critical risk factors that influence the uptake.	women (*n* = 2301). Age: ≥40 years	MM	83 (3.61%) have ever had mammography. Age and ethnic group were associated with mammography examination. Women aged _70 years had lower odds of having mammography examinations.	
Dadzi (2019) [30]	Volta region: Community setting.	Cross-sectional study	Assessed the awareness, knowledge, and practices of BSE as a method of prevention and early diagnosis of BC among reproductive-aged women in Akatsi South district in the Volta Region of Ghana.	Women (*n* = 385). Age: ≥8 years	BSE	The majority 279 (72.5%) reported not practicing BSE. Among those who practiced BSE, most 62(58.5%) reported performing BSE every month, 27(25.5%) examined the breast once every year. Reasons for not practicing are; do not know the techniques in BSE, do not have breast problems. Did not need BSE. Predictors of BSE were higher age with a lower practice of BSE, good knowledge of BC was a predictor of BSE.	Only 165 (43.3%) knew what BSE was. The source of knowledge was from the health care provider, relatives, friends, and books.
Fiador (2018)^*^ [31]	Greater Accra region: University community setting.	Cross-sectional study	to assess Knowledge, Attitude, and Practice of Breast self-examination (BSE) among female students of the University of Ghana	Female university students (343). Age: ≥18 years	BSE	61% practiced BSE. 16.9% practiced monthly. Predictors included increasing age, knowledge of BSE, level of Study. Barriers to BSE practice included having no reason, lack of knowledge, forgetfulness, fear to find a mass.	89.9% heard of BSE. Source of information; mass media, health professionals.
Ghansah (2019)^*^ [32]	Greater Accra region: Hospital setting.	Cross-sectional study	To assess the knowledge of BC, BCS practices, and health beliefs among female clinicians in two municipal hospitals in Accra.	Female clinicians (*n* = 283). Age: ≥18 years	BSE, CBE, and MM	Practiced BSE (77.4%,), CBE (21.6%) and Mammogram screening (1.1%). Medical doctors had the highest proportion of those who practice BSE 23(95.8%). General nurses are the second highest group with a proportion of 108 (81.2%) followed by the midwives with 81 (73.6%).	Most of the participants knew about BSE 95.4%). About 69% of the participants knew about CBE. Mammogram screening was known by about 54% of the participants.
Gyedu (2017) [33]	Ashanti region: Community setting.	Cross-sectional study	To characterize distinct differences in breast health engagement, perceptions, and participation of Ghanaian Muslim women compared with Christian women.	Women (*n* = 771). Age: ≥ 8 years	BSE and CBE	Four hundred nineteen women (54%) responded that they have been taught how to perform BSE, of which 356 (85%) answered that they have performed BSE at least once, 278 (66%) had performed BSE at least once per year, and 252 (60%) had performed BSE the recommended one time per month. Only 291 women (38%) had ever undergone CBE. Within groups, 217 Christian women (64%) had undergone CBE compared with only 74 Muslim women (17%; P,.001).	
Kudzawu (2016) [34]	Greater Accra region: Community setting.	Mixed method study	To determine the knowledge and practices of BSE among market women at Makola shopping mall in Accra.	Women (*n* = 170). Age: ≥18 years	BSE	Most women performed BSE but the minority who did not perform BSE was because they did not know how to do it, it was not necessarily religious faith. Most of the women performed BSE because they wanted to detect breast lumps early and fear death.	BSE awareness was high among women (158 (93%)). The source of awareness was health care providers and the media (radio and television).
Opoku (2012) [35]	Greater Accra and Brong Ahafo region (Sunyani).	Mixed-method study	Determined population-based rates of reported BCS and assessed breast cancer-related knowledge, attitudes, beliefs among Ghanaian women and explore their relation to screening practices in the study areas.	Women (BC patients and health women) (*n* = 474) Age:≥18 years	BSE, CBE, and MM	Self-reported BSE was 32%, CBE was estimated at 12% while mammogram was 2%. knowledge about BC, higher education was associated with BCS practices.	
Osei-Afriyie (2021) [36]	Volta region: University community setting.	Cross-sectional study	The study aimed to explore the awareness, risk factors, and self-reported screening practices of BC among female undergraduate students.	Female University students (n = 385). Age: 22 ± 2.78.	BSE, CBE, and MM	212 (55.1%) ever had one method of breast cancer screening. 164 (42.6%) practiced BSE, 39(10.2%) had undergone CBE, and 9 (2.3%) had mammography. Reasons for not practicing BSE was: not knowing how to perform it, having no family history of Bc, not being at risk of breast cancer. Women who were between 25 and 29 years old were 5.13 times more likely to perform regular BSE compared to those less than 20 years. Those who did not know their risk level were less likely to perform regular BSE.	
Sarfo (2013) [37]	Ashanti region: University community setting.	Cross-sectional study	To determine the knowledge, attitude, and practice of BSE among female university students.	Female university students (*n* = 250). Age: ≥18 years	BSE	76% performed BSE. 31% performed BSE monthly, 21% stated they performed BSE at random. 62% stated some days after menstruation. Reasons for not performing BSE: have no time, not necessary.	95% stated they knew breast cancer and BSE. 80% knew how to perform BSE. Source of knowledge: media.

*Legend***:** *BC* breast cancer, *BCS* breast cancer screening, *BSE* breast self-examination, *MM* mammography, *CBE* clinical breast examination

*Grey literature

### Practice of breast cancer screening

This review identified three methods of BCS (BSE, CBE, and mammography) utilized by Ghanaian women. Of the 14 studies, 12 reported evidence on the practice of BSE [25–28, 30–37]. Five studies provided evidence on the utilization of CBE [27, 32, 33, 35, 36]. The practice of BSE was reported a little above average among four studies with prevalence ranging from 63.3 to 77.3% [25, 28, 32, 37] while three studies recorded evidence of low practice [30, 35, 36] ranging from 27.5 to 42.6%. Among the three screening methods identified in this study, mammography had the lowest utilization rate of 13.6% [25] to as low as 1.1% [32]. Women that utilized CBE ranged from 10.2 to 38% among three studies [33, 35, 36]. Among women who practiced BSE, less than 50% of them performed BSE every month as recommended [25, 37]. Whiles studies conducted in the Volta region [30] and Ashanti region [33] of Ghana reported above 50% of once-monthly BSE practice. We also observed that some women performed BSE randomly [37] (see Tables 2, 3, and 4).

**Table 3:** Factors associated with breast cancer screening

Study	Factors associated with BCS	
	Significant	Not significant
Agyemang (2020) [13]	Cervical cancer screening, having at least a primary level education, and having ever participated in a club meeting.	Locality of residence.
Amenuke-Edusei (2020) [25]	Age, income, physicians’ recommendations.	Health insurance coverage.
Boafo (2019) [26]	Knowledge, Self-efficacy.	Susceptibility, severity, benefits, age.
Bonsu (2019) [27]	Contraceptive use, age, education, History of breast cancer and positive beliefs on breast cancer.	
Buunaaim (2020) [28]	Increased age, nurses, and market women.	
Calys-Tagoe (2020) [29]	Women aged _70 years, being self-employed, being an informal employee ethnic group.	
Dadzi, (2019) [30]	Age, knowledge of breast cancer.	Do not know the techniques in BSE, do not have breast problems. Did not need BSE.
Fiador (2018) [31]	Age, level of study, knowledge on BSE procedure, attitudes.	Not having a reason, lack of knowledge, forgetfulness.
Ghansah (2019) [32]		Medical doctors, nurses.
Gyedu (2017) [33]	Christian women.	
Kudzawu (2016) [34]		Did not know how Perform BSE, religious faith, wanted to detect breast lumps, fear death.
Opoku (2012) [35]	Knowledge about breast cancer, higher education.	
Osei-Afriyie (2021) [36]	Optimism regarding the breast cancer risk. Did not know their risk level, no religion.	Do not know how to perform BSE, have no family history of breast cancer, I am not at risk of breast cancer.
Sarfo (2013) [37]		Have no time, not necessary.

**Table 4:** Summary of key barriers and enabling factors of breast cancer screening uptake

Key findings	Included studies													
Barriers	Agyemang (2020) [13]	Amenuke-Edusei (2020) [25]	Boafo (2019) [26]	Bonsu (2019) [27]	Buunaaim (2020) [28]	Calys-Tagoe (2020) [29]	Dadzi (2019) [30]	Fiador (2018) [31]	Ghansah (2019) [32]	Gyedu (2017) [33]	Kudzawu (2016) [34]	Opoku (2012) [35]	Osei-Afriyie (2021) [36]	Sarfo (2013) [37]
Did not know how to perform BSE	–	**–**	–	–	–	–		–	–	–	*	–	*	–
Not at risk of BC	–	–	–	–	–	–	**–**	–	–	–	–	–	*	–
No breast problem/signs of BC	–	*	–	–	–	–	*	–	**–**	–	–	–	–	–
No family history of BC	–	–	–	–	–	–	**–**	–	–	–	–	–	*	–
No physician recommendation	–	*	–	–	–	–	–	–	**–**	–	–	–	–	–
Lack of awareness of BCS	–	*	–		–	–		*	–	–	–	–	–	–
Not necessary	–	–	–	–	–	–	**–**	–	–	–	*	–	–	*
Religious faith	–	–	–	–	–	–	–	–	–		*	–	–	–
Younger age	–	–	–	–	–	–	*	–	–	–	–	–	–	–
Older age	–	–	–	–	–	*	–	*	–	–	–	–	–	–
Fear of finding a mass	–	–	–	–	–	–	–	*	–	–	–	–	–	–
**Enabling factors**														
Good knowledge	–	–	*	–	–	–	*	–	–	–	–	*	–	–
High education	*	–	–	*	–	–	–	–	–	–	–	*	–	–
For early detection	–	–	–	–	–	–	–	–	–	–	*	–	–	–
Routine medical checkup	–	*	–	–	–	–	–	–	**–**	–	–	–	–	–
High self-efficacy	–	–	*	–	–	–	–	–	–	–	–	–	–	–
Increasing age	–	*			*									
Monthly income	–	*	–	–	–	–	–	–	–	–	–	–	–	–
Physician recommendation	–	*	–	–	–	–	–	–	–	–	–	–	–	–
Prior cervical screening	*	–	–	–	–	–	–	–	–	–	–	–	–	–
Contraceptive use	–	–	–	*	–	–	–	–	–	–	–	–	–	–
Fear of death	–	–	–	–	–	–	–	–	–	–	*	–	–	–
Age at menarche	–	–	–	*	–	–	–	–	–	–	–	–	–	–

*Legend*: *BC* breast cancer, *BCS* breast cancer screening *Factor (s) identified

### Barriers to breast cancer screening practices

Among the studies included in this review, several barriers to BCS practice were identified among women in different regions across Ghana. The most common reason why women did not perform BSE was due to the lack of technique to perform BSE [30, 34, 36]. Some women in the Volta region who reported not having breast cancer risk or did not know their risk level were less likely to perform BSE [36]. We also observed in some studies that women who did not have any breast problems opted not to have BCS [25, 30]. Some women did not practice BSE because they saw the procedure to be unnecessary [34, 37] and some also felt they had no time [37]. It was also observed that lack of awareness of BCS was a barrier to women’s uptake of any form of screening method [25]. Women who did not get a recommendation from a physician did not practice BCS [25]. We also observed that women aged 70 years and above had lower odds of having a mammogram [29] (see Tables 2, 3 and 4).

### Predictors of breast cancer screening practices

This review unraveled several predictors of BCS practices among women in Ghana but the most common predictor among the studies was the woman having good knowledge of breast cancer or the screening practice [26, 27, 35]. We also observed that the increasing age of women [25, 28, 36] and higher educational level [27, 35] were significant contributory factors to the utilization of BCS in Ghana. A study conducted in Greater Accra indicated women underwent mammography screening because of routine medical check-ups [25]. It was also observed that the history of BC was also identified to be associated with the uptake of BCS among women in the Ashanti region [27], while in the Greater Accra region due to fear of death some women utilized BCS in order to ensure early detection and treatment [34]. A study by Amenuke-Edusei and Birore [25] uncovered that physician recommendation and monthly income were positive influencing factors for women utilizing mammography in the Greater Accra region. Women who had positive beliefs about breast cancer [27] and higher self-efficacy [26] were most likely to utilize BCS. A study by Agyeman et al. [13] reported that women who have ever had cervical cancer screening were more likely to screen for breast cancer (see Tables 2, 3 and 4).

## Discussion

This scoping review aimed to explore evidence on Ghanaian women’s knowledge and practice of BCS to inform policy and public health strategies to improve screening practices. The review revealed varied knowledge levels and practices of BCS among women across a few regions in Ghana. The knowledge level of women on BCS was exponentially high, especially in BSE practice. The finding of this study is similar to a study conducted in Nigeria where 97% of women were aware of BSE [38] but different from a study conducted in Ethiopia where only 6.6% of women heard of BCS practice and 5.2% of them knew about BCS [39]. This divergence could be due to the mass campaign carried out within Ghana on BCS uptake as most of the studies reported that women’s main source of knowledge was from the healthcare providers and the mass media.

Although we observed a high awareness/knowledge level of Ghanaian women on BCS especially BSE, this was not much reflective on the practice level of women on the various methods of BCS. We observed a little above average and low practice rates among women in Ghana. One will assume that the high awareness level seen among women would have culminated into high practice levels but that has not been the case in this review. Screening mammography was observed to be very low among women with 13.6% [25] to as low as 1.1% [32]. The evidence reported on CBE also shows a low screening rate among women in Ghana. Our study finding is not different from a recent population-based study conducted in four sub-Saharan African countries where the overall prevalence of BCS was 12.9% ranging from 5.2% in the Ivory Coast to 23.1% in Namibia [40]. Evidence from prior cohort studies based on mammography screening programs among women aged 50-69 years in high-income countries indicates a 23% reduction in breast cancer-related mortalities [41]. Several other studies have also reported screening via mammography reduces breast cancer-related deaths by 15–30% [42–44]. Several empirical evidence has also proven that CBE and BSE ensure early detection, diagnosis, and treatment of breast cancer among women [45, 46]. As empirical evidence shows that BCS aids in the reduction in the mortalities related to breast cancer, intervention programs must be initiated to help improve the screening rate among women in Ghana to ensure early detection, diagnosis, and treatment.

We identified several factors contributing to the low prevalence rate of BCS among women in Ghana. The most identified barriers were lack of technique to practice BSE, having no breast problem, and not having BC risk. Identifying these barriers and several others will inform health care professionals and other important stakeholders on the important public health strategies and interventions to implement in order to mitigate these stumbling blocks to women patronizing BCS uptake.

Though some BCS barriers were identified, this review uncovered several factors predicting the utilization of BCS uptake among women. The prominent enabling factors include good knowledge of BCS, high education level, increasing age, physician recommendation, etc. This finding is in line with a recent study conducted in 14 low-resource countries where a higher educational level of women was found to be associated with the uptake of BCS [47]. Women with high education are more aware of health complications and adverse effects of diseases and, therefore, have regular medical check-ups as preventive measures, including BCS services. Therefore, interventions targeted to increase BCS rates may emphasize specifically on those with low educational background or may concentrate on increasing women’s health education and awareness levels to ensure population-level increases in BCS services. Another important enabling factor of BCS uptake is the increasing age of women. Contradicting this finding some studies rather found the younger age to be an enabling factor for BCS. This finding shows that educational and interventional programs should be equally targeted at both the young and old in order to promote the utilization of BCS services. We also identified that women with good knowledge of BCS were more likely to utilize BCS services. Hence improving women’s knowledge of the various BCS methods will go a long way to improve their screening behaviors.

We observed that women’s household monthly income or good socioeconomic status was a predictor of women undergoing BCS services. This finding is congruent with a study conducted in low and middle-income countries where women in the wealthier quintile were more likely to undergo BCS services than the poor [47]. Therefore, the conclusion that women with the wealthiest economic status are significantly more likely to utilize BCS services in low-resource countries also aligns with previous findings [47–49]. Physicians’ recommendations were also identified as an important factor for women utilizing BCS. Our finding is consistent with prior studies that reported that physician recommendations were positively associated with BCS (mammography) uptake [50, 51]. This shows the critical role physicians play in ensuring women undergo BCS, especially mammography screening.

### Implications for practice, policy and research

The study revealed different prevalences, barriers, and enabling factors of BCS across the various regions. The low rate of BCS practices especially CBE and mammography screening among women across the various regions raises concerns about the health system’s readiness to address this significant back-drop. Mammography has been recognized as a gold standard for BCS in high-income countries, based on previous randomized controlled trials that observed significant reductions in the mortality rates among women aged 50 and above who participated in the organized mammography screening programs [4]. It is therefore imperative for effective awareness creation on the importance of mammography screening and its uptake. Organized mass mammography screening in various regions is needed to improve women screening uptake.

CBE is a relatively simple and inexpensive method for early detection of breast tumors [41] and can adequately be performed by trained, non-medical health workers [52]. Providing CBE at the community level and strengthening the health care system to provide these services is needed.

Ghana initiated a national strategy for cancer control for the period of 2012–2016 where the program was to create breast awareness, provide education on breast cancer prevention and BSE among girls older than 18 years. Also, health professionals were to be trained every 2 to three years to offer CBE in health facilities across the country [53, 54]. This national strategic plan has not yet been implemented [54], therefore, there is the need for the government of Ghana to implement this national policy to improve the screening practices of women and to reduce the burden of breast cancer.

Further research is recommended to assess the attitude of Ghananian women towards the various methods of BCS as the studies included in this review did not assess women’s attitudes towards screening. We recommend that the implementation of policies and public health intervention strategies targeted at improving women’s knowledge and practice towards BCS is imperative.

### Strengths and limitations

The strength of this review is the strict adherence to the guideline provided by Arskey and ‘O’Malley’s framework for scoping reviews [21]. Another strength of this review is the use of a comprehensive, systematic search strategy in different databases to identify relevant studies. Even though methodological rigor was applied, this review had some limitations. First, the six regions captured in this review might not be representative of the entire 16 regions in Ghana so the findings should be interpreted with caution. Also, the authors may have unintentionally omitted relevant studies from this review although extensive database and hand searches were conducted.

## Conclusion

The findings from this review showed great heterogeneity in BCS prevalence across the regions in Ghana. BCS services are very important for women as it helps to reduce the burden of breast cancer where it is poorly documented especially in low and middle-income countries. Despite the benefits of BCS services, the prevalence of BCS across the regions of Ghana is very low due to some varied barriers from the different regions. The findings further showed that good knowledge of BCS, higher educational level, increasing age, physician recommendation, and household monthly income were enabling factors for BCS uptake.

## Supplementary Information

**Additional file 1.**

## Data Availability

The datasets used and/or analyzed during the current study are available from the corresponding author on reasonable request.

## Abbreviations

BCS: Breast Cancer Screening
BSE: Breast self-examination
CBE: Clinical breast examination
MM: Mammography
LMICs: low-middle-income countries
WHO: World Health Organisation
SDG: Sustainable Development Goal
