# Supplementary material for: Mapping evidence on knowledge of breast cancer screening and its uptake among women in Ghana: a scoping review
Source: BMC Health Serv Res. 2022 Apr 20;22:526. doi: 10.1186/s12913-022-07775-z (PMC9022278; doi:10.1186/s12913-022-07775-z)
Supplement: Supplementary file 1 — Additional file 1. [file 12913_2022_7775_MOESM1_ESM.pdf]

Table S1: sample of search strategies

Databases	No	Search terms	Results
PubMed			
	1	("Breast Neoplasms"[MeSH Terms] OR "Breast Neoplasms"[All Fields] OR "Breast cancer"[All Fields] OR "Breast Neoplasia"[All Fields] OR "Breast Tumor"[All Fields]) AND (2012/1/1:2021/9/30[pdat])	185,340
	2	("Early Detection of Cancer"[MeSH Terms] OR "Mass Screening"[MeSH Terms] OR "Breast cancer early detection"[All Fields] OR "Breast cancer screening"[All Fields] OR "Breast screening"[All Fields] OR "Cancer screening"[All Fields] OR "Early diagnosis of cancer"[All Fields] OR "Cancer early diagnosis"[All Fields] OR "Screen-and-treat"[All Fields] OR "Screening"[All Fields] OR "breast self-examination"[All Fields] OR "Clinical breast examination"[All Fields] OR "Breast examination"[All Fields] OR "mammography"[MeSH Terms] OR "mammography"[All Fields] OR "mammogram"[All Fields] OR "mammograms"[All Fields] OR "mammography"[MeSH Terms] OR "mammography"[All Fields] OR "mammographies"[All Fields] OR "mammography s"[All Fields]) AND (2012/1/1:2021/9/30[pdat])	368,621
	3	("knowledge"[MeSH Terms] OR "knowledge"[All Fields] OR "knowledge s"[All Fields] OR "knowledgeability"[All Fields] OR "knowledgeable"[All Fields] OR "knowledgeably"[All Fields] OR "knowledges"[All Fields] OR "practicability"[All Fields] OR "practicable"[All Fields] OR "practical"[All Fields] OR "practicalities"[All Fields] OR "practicality"[All Fields] OR "practically"[All Fields] OR "practicals"[All Fields] OR "practice"[All Fields] OR "practice s"[All Fields] OR "practiced"[All Fields] OR "practices"[All Fields] OR "practicing"[All Fields]) AND (2012/1/1:2021/9/30[pdat])	1,220,490
	4	("ghana"[MeSH Terms] OR "ghana"[All Fields] OR "ghana s"[All Fields] OR "ghanaian"[All Fields] OR "ghanaians"[All Fields]) AND (2012/1/1:2021/9/30[pdat])	13,688
	5	**#1 AND #2 AND #3 AND #4**	24
**Web of science**	1	TS(‘Breast cancer’ OR ‘Breast Neoplasia’ OR ‘Breast Neoplasms’ OR ‘Breast Neoplasm’ OR ‘Breast Tumor’)	328,176
	2	TS("Early Detection of Cancer" OR "Mass Screening" OR "Breast cancer early detection" OR "Breast cancer screening" OR “Breast screening” OR "Cancer screening" OR "Early diagnosis of cancer" OR "Cancer early diagnosis" OR “Screen-and-treat” OR "Screening" OR	387,081

		"breast self-examination" OR “Clinical breast examination” OR “Breast examination” OR Mammogram OR Mammography)	
	3	TS (Knowledge OR Practice)	1,744,482
		TS (Ghana OR Ghanaian)	19,764
	4	**#1 AND #2 AND #3 AND #4**	**21**
**CINAHL**	1	("Breast cancer" OR "Breast Neoplasia" OR "Breast Neoplasms” OR "Breast Neoplasm" OR “Breast Tumor”)	61,392
	2	("Early Detection of Cancer" OR "Mass Screening" OR "Breast cancer early detection" OR "Breast cancer screening" OR "Breast screening" OR "Cancer screening" OR "Early diagnosis of cancer" OR "Cancer early diagnosis" OR "Screen-and-treat" OR "Screening" OR "breast self-examination" OR "Clinical breast examination" OR "Breast examination" OR Mammogram OR Mammography)	124,362
	3	(Knowledge OR Practice)	531,328
		(Ghana OR Ghanaian)	4,145
	4	**#1 AND #2 AND #3 AND #4**	5
**EMBASE**	1	('breast cancer'/exp OR 'breast neoplasia' OR 'breast neoplasms'/exp OR 'breast neoplasm' OR 'breast tumor'/exp) AND [1-1-2012]/sd NOT [1-10-2021]/sd	322542
	2	('early detection of cancer'/exp OR 'mass screening'/exp OR 'breast cancer early detection' OR 'breast cancer screening' OR 'breast screening' OR 'cancer screening'/exp OR 'early diagnosis of cancer' OR 'cancer early diagnosis' OR 'screen-and-treat' OR 'screening'/exp OR 'breast self-examination'/exp OR 'clinical breast examination'/exp OR 'breast examination'/exp OR 'mammogram'/exp OR 'mammography'/exp) AND [1-1-2012]/sd NOT [1-10-2021]/sd	417160
	3	('knowledge'/exp OR 'practice'/exp) AND [1-1-2012]/sd NOT [1-10-2021]/sd	104566
		('ghana'/exp OR 'ghanaian'/exp) AND [1-1-2012]/sd NOT [1-10-2021]/sd	9820
	4	**#1 AND #2 AND #3 AND #4**	9
**PsycINFO**	1	("Breast cancer" OR "Breast Neoplasia" OR "Breast Neoplasms" OR "Breast Neoplasm" OR "Breast Tumor")	6,999
	2	("Early Detection of Cancer" OR "Mass Screening" OR "Breast cancer early detection" OR "Breast cancer screening" OR "Breast screening" OR "Cancer screening" OR "Early diagnosis of cancer" OR "Cancer early diagnosis" OR "Screen-and-treat" OR "Screening" OR "breast	47,794

		self-examination" OR "Clinical breast examination" OR "Breast examination" OR Mammogram OR Mammography)	
	3	(Knowledge OR Practice)	415,949
	4	(Ghana OR Ghanaian)	4,286
	5	#1AND #2 AND #3 AND #4	3
